# A Systematic Review of Eye-Tracking Studies of Construction Safety

**DOI:** 10.3389/fnins.2022.891725

**Published:** 2022-04-26

**Authors:** Baoquan Cheng, Xiaowei Luo, Xiang Mei, Huihua Chen, Jianling Huang

**Affiliations:** ^1^Department of Engineering Management, School of Civil Engineering, Central South University, Changsha, China; ^2^Department of Architecture and Civil Engineering, City University of Hong Kong, Kowloon, Hong Kong SAR, China; ^3^Anhui BIM Engineering Center, School of Civil Engineering, Anhui Jianzhu University, Hefei, China

**Keywords:** eye-tracking, construction safety, hazard recognition, review, neuromanagement in engineering

## Abstract

Safety is the most important concern in the construction industry, and construction workers’ attention allocation is closely associated with their hazard recognition and safety behaviors. The recent emergence of eye-tracking techniques allows researchers in construction safety to further investigate construction workers’ visual attention allocation during hazard recognition. The existing eye-tracking studies in construction safety need to be comprehensively understood, to provide practical suggestions for future research and on-site safety management. This study aims to summarize previous studies on the application of eye-tracking techniques to the construction safety context through a systematic literature review. The literature search and study selection process included 22 eligible studies. Content analysis was then carried out from participant selection, device selection, task design, area of interest determination, feature extraction, data analysis, and main findings. Major limitations of the existing studies are identified, and recommendations for future research in theoretical development, experiment improvement, and data analysis method advancement are proposed to address these limitations. Even though the application of eye-tracking techniques in construction safety research is still in its early stage, it is worth future continuous attention because relevant discoveries would be of great significance to hazard control and safety management in the construction industry.

## Introduction

The construction industry is considered one of the most hazardous industries because of its complex and dynamic workplaces. The International Labor Organization (ILO) estimated that at least 60,000 people lose their lives in construction safety accidents each year, equating to one fatality every 10 min. In industrialized countries, the construction industry accounts for as many as 25–40% of workplace fatalities despite the sector employing only 6–10% of the workplace (International Labour Organization, 2005). Construction workers suffered a 3–4 times higher workplace fatality rate than workers in other sectors (International Labour Organization, 2015). In Hong Kong, of the 25 fatal workplace fatalities in 2014, 20 accidents (accounting for 80%) occurred in the construction industry (Hong Kong OSHC, 2015). According to Safe Work Australia (SWA), the construction industry recorded 12% of workplace fatalities in Australia (Safe Work Australia, 2015). The annual workplace fatalities in the construction industry accounted for about 21% of all workplace fatalities in 2020, according to the United States Bureau of Labor Statistics (BLS) (U.S. Bureau Of Labor Statistics, 2020). In the mainland, China, there are 734 cases of safety accidents occurred in the construction industry occurred in 2021, with 840 workers lost their lives. Therefore, construction safety has become a global concern. Enhancing hazard control and safety management has been a top priority in the construction industry worldwide.

Safety management has attracted wide attention from scholars worldwide. Heinrich (1941) indicated that hazardous working conditions in the workplace and unsafe behaviors are two direct causes of safety accidents in the construction industry. Therefore, successfully identifying these environmental conditions and unsafe behaviors, i.e., hazard recognition, is the foundation of improving construction safety (Haslam et al., 2005). Unfortunately, about 57% of hazards in construction sites remain unrecognized (Perlman et al., 2014). Some studies applied survey-based methods including site surveys, questionaries, and expert interviews, to investigate hazard recognition and related factors (Gürcanlı et al., 2015; Pandit et al., 2019; Ma et al., 2021). However, these methods are likely to cause subjective bias and memory bias. To overcome shortcomings of pure survey-based studies, some studies combined some simple hazard-recognition tasks in experiments (Perlman et al., 2014; Albert et al., 2020; Han et al., 2021; Wolf et al., 2022). Participants are asked to identify potential hazards based on photos or immersive virtual environments of construction sites. These studies provide an objective tool to assess the construction workers’ hazard recognition performance. However, in-time hazard recognition and responses form a complicated and multifaceted cognitive process. These studies failed to understand the mechanism of construction workers’ hazard recognition from the cognitive level.

Neuromanagement in engineering, which integrates research approaches in neuroscience into engineering management (Yu et al., 2022), provide a powerful and advanced tool for human cognition and behavior research besides hazard recognition of construction workers. Emerging neuroscience techniques including eye-tracking, electroencephalogram (EEG) (Cheng et al., 2022; Saedi et al., 2022), functional near-infrared spectroscopy (fNIRS) (Zhou et al., 2021). EEG is a diagnostic imaging technique used to obtain information about brain activities by detecting the voltage variations induced by neurons on the scalp cortical surface (Jeon and Cai, 2021; Ke et al., 2021; Peng et al., 2022). fNIRS is an optical brain monitoring technique which uses near-infrared spectroscopy to estimate cortical hemodynamic activity which occur in response to neural activities (Sun and Liao, 2019; Liao et al., 2021). These two methods can both capture rich cognitive information in brain activities (Fan et al., 2021). Therefore, they have been applied to explore the cognitive process of construction workers during hazard recognition. Although EEG and fNIRS provide researchers deep insight into construction workers’ cognitive process, they cannot capture visual attention, which is essential for hazard recognition of construction workers (Zhang et al., 2019). Eye-tracking is a technique for monitoring the point of fixation (where one is looking) or the motion of an eye in relation to the head (Klaib et al., 2021). It enables to capture the individual visual attention (Günther et al., 2021) and has been widely used in research in the fields of marketing (Rusnak, 2021; Guo et al., 2022), transportation (Topolšek et al., 2016; Ahlström et al., 2021), medicine (Tien et al., 2014), criminalistics (Gehrer et al., 2021), professional education (Salehi et al., 2018), advertisements (Yen and Chiang, 2021), etc. In the construction industry, eye-tracking also allows researchers and safety managers to deeply understand the process and mechanism of construction workers’ hazard recognition and responses by observing and analyzing their visual patterns under different circumstances, and then take positive measures to improve construction safety management (Hasanzadeh et al., 2019; Chong et al., 2021). Therefore, eye-tracking has emerged as one of the most rapid-developing technologies in construction safety studies.

Despite the potential of applying eye-tracking techniques in construction safety management, the body of knowledge lacks a systematic review in this field. With an increasing number of studies on this subject, such a systematic review enables organizing their research topics, methodologies, and findings. It also aids in identifying the research gaps and thus develops an actionable reference to pathways for future research. In addition, the examination of the scholarly works sheds light on the researchers and professionals engaged in the implementation of eye-tracking in construction safety, paving the way for future cooperation to share knowledge and extend the application of eye-tracking techniques in construction safety management. Therefore, the present review summarized existing studies on the application of eye-tracking techniques to the construction safety context. By conducting a comprehensive search and evaluation of involved studies, we aimed to provide recommendations for future research and safety management practices in the construction industry.

## Research Methodology

**FIGURE 1:**
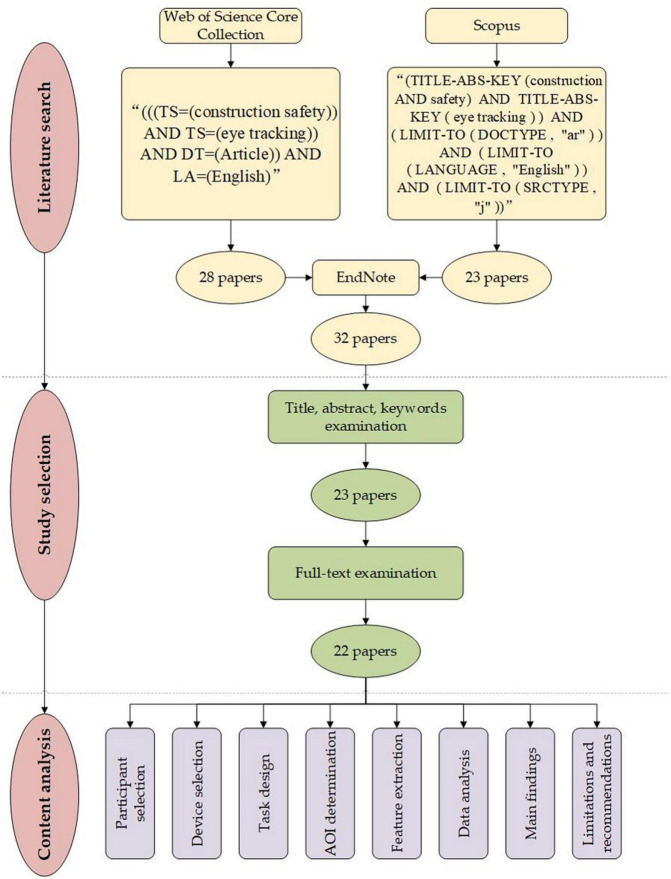
Overview of review methodology.

The systematic review refers to a type of review that applies repeatable analytical methods to collect secondary data from existing literature and analyze it. These reviews are usually conducted to offer an exhaustive summary of current evidence related to a research question (Armstrong et al., 2011). In this study, a systematic review enables comprehensively evaluating the existing eye-tracking studies of construction safety. Therefore, it is adopted. The research methodology is developed based on the earlier guidance on the conduct of systematic reviews articulated by de Araújo et al. (2017). As shown in Figure 1, the methodology of the review comprises three steps: literature search, study selection, and content analysis.

### Literature Search

An exhaustive search of published literature was conducted according to the titles, abstracts, and keywords in two of the most reputable academic databases, Web of Science Core Collection (WoS), and Scopus. These two databases are among the largest online academic sources that cover more journals and recent publications, and all covered journals are reviewed for sufficiently high quality each year. Because the present review focused on eye-tracking and its application in construction safety, the search scope considered two aspects: construction safety and eye-tracking. In addition, this review only considered research papers published in peer-reviewed journals written in English. The review papers, book chapters, conference papers, and papers in other languages were excluded. No time limitations have been set during the literature search. Therefore, the retrieval codes for WoS and Scopus were “(((TS = (construction safety)) AND TS = (eye tracking)) AND DT = (Article)) AND LA = (English)” and “(TITLE-ABS-KEY (construction AND safety) AND TITLE-ABS-KEY (eye tracking)) AND (LIMIT-TO (DOCTYPE, “ar”)) AND (LIMIT-TO (LANGUAGE, “English”)) AND (LIMIT-TO (SRCTYPE, “j”)),” respectively. As a result, 28 and 23 articles were retrieved from WoS and Scopus. All identified literature from two databases was imported into Endnote software to eliminate duplicates, resulting in 32 articles.

### Study Selection

This review was scoped on the application of eye-tracking in construction safety. Even though the appreciate structural retrieval codes have been used, original results still included some literature that matches the retrieval codes but is not closely related to the topic. A two-round screening process was used to further select papers for inclusion. The first round excluded irrelevant papers based on their titles, abstracts, and keywords. The remaining papers were then subjected to a full-text examination in the second round to determine their viability. Some papers cited by the papers in the second round may fit into the present review’s interest but were not found in the original database search. These papers would also be added for full-text examination.

Of the 32 articles identified in the initial literature search, nine irrelevant papers were excluded in the first round of screening because seven papers were not related to construction safety, and two papers did not use an eye-tracking system. Through the second round of screening, we found a paper that used an eye-tracking sensor in its experiment but did not analyze the eye-tracking data (Kim et al., 2021b). Therefore, this paper was also excluded. No references from the biographies were added through snowball search. The screening process finally retained 22 papers for the content analysis.

### Content Analysis

Study characteristics, such as publication information, participants, device, experimental tasks, eye-tracking indicators, data analysis methods, and main findings of identified studies, were summarized to provide basic details of the included papers. Hazard recognition is the foundation to ensure construction safety. Therefore, one major issue in the present review was to find out how hazard recognition performance can be evaluated with eye-tracking data. Another important concern of the present review was the changes in eye-tracking indicators and hazard recognition performances in relation to the work environment, worker personalities, and other factors. A summary of findings related to the second issue can provide valuable information for construction safety management practice. The limitations of current studies were also summarized to provide recommendations for future studies.

## Results

### Descriptive Analysis

A total of 22 papers about the application of eye-tracking in construction safety are identified through literature search and further study selection. Further information regarding the features of the included studies can be found in Table 1. The number in brackets in the participant row means valid samples. As for their annual distribution, it can be found that the first related paper was published in 2016, and after that, the annual number of eye-tracking studies of construction safety increased generally. In 2021, the annual publication number reached seven. This suggests that applying eye-tracking in construction safety management is an emerging topic that attracts attention from researchers. In addition, almost all the papers are published in civil engineering journals such as Journal of Construction Engineering and Management (seven papers, accounting for 32%), Automation in Construction (two papers, accounting for 9%), KSCE Journal of Civil Engineering (one paper, accounting for 4%), and Journal of Management in Engineering (one paper, accounting for 4%). There are also some papers published in journals in computer science, operational research and management science, and public, environmental, and occupational health. This indicated that the application of eye-tracking in construction safety management is cross-disciplinary research.

### Participant Selection

Sufficient and representative participants are essential to get high-quality eye-tracking data. A sufficiently large participant size reduces the accident errors caused by individual variations of participants and strengthens the dependability of conclusions. The valid participant size in eye-tracking studies of construction safety ranges from 6 to 55, with an average value of 31 and a median value of 30. Since eye-tracking studies are experimental research, the participant size is challenging to be as large as survey-based research. Previous studies suggested that 30 is an ideal participant size in eye-tracking studies (Pernice and Nielsen, 2009), proving that participant sizes in most reviewed studies are rational. It should be noted that eight studies (accounting for 36%) excluded some participants, and the average rejection rate is about 22%. Invalid participants are rejected for a variety of reasons such as unacceptable levels of calibration on the eye tracker (Hasanzadeh et al., 2017a,b, 2018; Hasanzadeh et al., 2019), univariate outliers (Hasanzadeh et al., 2017a), interruptions during eye-tracking experiments (Hasanzadeh et al., 2017a), abnormal or incorrectible-to-normal vision (Park et al., 2022), failures in pre-experiments (Liao et al., 2019; Sun et al., 2020), and uncooperative participants (Cheng et al., 2021; Park et al., 2022). This indicates that to ensure the sufficient experiment size of participants, the researchers should recruit more participants as appropriate. In addition, pre-experiment is effective in preventing invalid participants’ influence on the results.

Of the 22 eye-tracking studies of construction safety, only 11 studies (accounting for 50%) selected construction workers as all participants, and others partially or totally selected students instead. Hazards recognition and safety behaviors of construction workers are significantly affected by human factors like age, work experience, personality, etc. (Siu et al., 2003; Dzeng et al., 2016; Liang et al., 2022). Selecting students as participants instead of construction workers help to control irrelevant variables during experiments. For example, Han et al. (2020) investigated how site conditions affect construction workers’ hazard recognition performance. Personal characteristics like experience should be consistent among participants rather than acting as an additional independent variable. Therefore, they recruited students with similar backgrounds for the hazard recognition experiment. In addition, Dzeng et al. (2016) found that it is more difficult to recruit construction worker participants than student participants. However, it should be noted that construction workers have apparent differences in group characteristics with students. Therefore, whether the findings and conclusions drawn by students suit construction workers needs more evaluation.

**TABLE 1:** Summary information of existing eye-tracking studies in construction safety.

References	Journal	Participants	Device	Experiment task	Eye-tracking indicator	Analysis method	Main findings
Dzeng et al. (2016)	Safety Science	Ten construction workers and 15 students	ViewPoint EyeTracker	Visually search hazards in four virtual construction sites	Fixation count and fixation sequence	Independent and paired *t*-test	While expertise aided experienced construction workers in evaluating visible and hidden hazards substantially quicker than fresh workers, it had little effect on their ability to identify hazards accurately.
Hasanzadeh et al. (2017b)	Journal of Management in Engineering	31 construction workers (27)	SR Research EyeLink II	Visually search hazards in 35 given images of site scenes	Time to first fixation, fixation time ratio, and run count	Permutation simulation	Tacit safety knowledge obtained from work experience and injury exposure significantly improve construction workers’ hazard recognition.
Hasanzadeh et al. (2017a)	Journal of Construction Engineering and Management	31 construction workers (21)	SR Research EyeLink II21qa	Visually search hazards in 35 given images of site scenes	Fixation count, fixation time ratio, and run count	Analysis of variance and discriminant analysis	Hazard recognition skills significantly affect construction workers’ visual search strategies, and thus eye-tracking indicators can be applied to identify individuals with poor hazard recognition skills.
Jeelani et al. (2017a)	Journal of Construction Engineering and Management	Eight construction workers	EyeTech VT3	Visually search hazards in 24 given images of site scenes	Fixation time	Interrupted longitudinal regression analysis	The eye-tracking system can provide feedback for hazard recognition performance and develop an efficient personalized safety training intervention for construction workers.
Hasanzadeh et al. (2018)	Journal of Construction Engineering and Management	14 students (11)	Tobii Pro Glasses 2	Walk along a path in the presence of other workers who are conducting their normal activities on the job site	Time to first fixation, fixation time, fixation count, and run count	Permutation simulation	Construction workers with higher situation awareness periodically looked sown and scanned ahead to remain fully aware of the environment and its associated hazards when walking.
Jeelani et al. (2018)	Automation in Construction	Five students and one construction professional	Tobii Pro Glasses 2	Visually search hazards in real construction sites	Fixation time, fixation count, search duration, mean fixation duration, visual attention index, and fixation time ratio	Descriptive statistical comparisons	Integrating computer-version and eye-tracking enables automating and scaling personalized safety training.
Hasanzadeh et al. (2019)	Journal of Construction Engineering and Management	31 construction workers (28)	SR Research EyeLink II21qa	Visually search hazards in 35 given images of site scenes	Fixation count and run count	Correlational analysis and permutation analysis	Extraversion, conscientiousness, and openness to experience significantly affect construction workers’ attention allocations and workers who are less extroverted, more conscientious and more open to experience have better performance in hazard recognition.
Jeelani et al. (2019)	Journal of Construction Engineering and Management	23 construction workers	EyeTech VT3	Visually search hazards in 12 given images of site scenes	Search duration, fixation count, fixation time, mean fixation duration, fixation spatial density, fixation count ratio, fixation time ratio, and saccade velocity	Regression analysis	Visual search patterns are predictive of hazard recognition performance of construction workers, and personalized safety training intervention can enhance their hazard recognition ability by improving their visual search patterns.
Li et al. (2019)	Automation in construction	12 construction workers	Pupil Lab	Operate an excavator while recognizing and responding to potential hazards that may cause collisions in a simulation system	Blink count, blink duration, pupil diameter, percent change in pupil diameter, fixation time, and fixation count	Analysis of variance and spearman correlation analysis	The hazard recognition ability of construction workers decreases with the mental fatigue level increasing, and it is associated with the changes in the distribution of construction workers’ visual patterns.
Liao et al. (2019)	International Journal of Occupational Safety and Ergonomics	42 construction workers (32)	SMI iView X	Visually search hazards in eight given images of site scenes	Fixation time ratio, and mean fixation duration	Logistic regression analysis	The effect of strengthened working memory on the detection rate through increased search efficiency is more apparent in high visual clutter.
Sun and Liao (2019)	Safety Science	48 students	Tobii Pro Glasses 2	Visually search hazards in a structural laboratory	Time to the first fixation	Analysis of variance and discriminant analysis	Hazard recognition ability can be better assessed by comprehensively considering eye-tracking and near-infrared spectroscopy (NIRS) indicators.
Xu et al. (2019)	Safety Science	47 students	Tobii Pro Glasses 2	Visually search hazards in a structural laboratory	Fixation sequence	Hierarchical clustering	Successful hazard recognitions have similar visual search patterns.
Han et al. (2020)	Journal of Construction Engineering and Management	55 students	Tobii T60XL	Visually search hazards in 20 given images of site scenes	The accuracy rate of the first fixation, fixation count, intersection coefficient, search duration, and fixation count in the attention center zone	Descriptive statistical comparisons	More distinct hazards and a tidy site reduce construction workers’ cognitive loads in hazard recognition, while site brightness has positive and negative effects.
Sun et al. (2020)	International Journal of Occupational Safety and Ergonomics	42construction workers (30)	SMI iView X	Visually search hazards in 15 given images of site scenes	Fixation time	Logistic regression analysis	Navigated safety inspection can improve hazards recognition effectiveness, and efficiency in scenes with high and medium visual clutters. A random search model can describe the visual patterns of construction workers in hazard recognition tasks.
Cheng et al. (2021)	International Journal of Environmental Research and Public Health	85 construction workers (55)	Tobbi Pro Fusion	Visually search hazards in 120 given images of site scenes	Fixation sequence	Temporal qualitative analysis	In the potential electrical contact hazards, the intersection of the energy-releasing source and wire is the cognitively driven visual area that construction workers are likely to prioritize. And as for PPE-related hazards, scene-related, and norm-guided are two different visual strategies generalized according to the workers’ visual cognitive logic.
Chong et al. (2021)	KSCE Journal of Civil Engineering	47 students	Tobii Pro Glasses 2	Visually search hazards in a structural laboratory	Fixation sequence	Crisp-set qualitative analysis	Electricity-related hazards should be recognized based on object identification, while struck-by hazards should be identified based on the objects and their pivot points or probable movement trajectories.
Comu et al. (2021)	Advanced Engineering Informatics	Five engineers, five laborers workers, and five students	Tobii Pro X2–30 Hz	Receive two different forms of safety training (i.e., a traditional presentation with 11 slides and a construction task level serious game)	Time to first fixation, and fixation time	Analysis of variance	The effect of safety training is significantly affected by the background of trainee construction workers and training methods.
Kim et al. (2021b)	Advanced Engineering Informatics	32 students	-	Clean the road while recognizing and avoiding struck-by hazards caused by surrounding construction equipment in an immersive virtual construction site	Pupil diameter and saccadic velocity	Analysis of variance and support vector machine	There exist significant differences in electrocardiogram (ECG) and eye-tracking indicators of construction workers between recognizing and ignoring hazards, and thus these biosignals can be applied for inattentiveness identification.
Liao et al. (2021)	Safety Science	48 students	Tobii Pro Glasses 2	Visually search hazards in a structural laboratory	Pupil diameter	Analysis of variance, permutation analysis, and correlation analysis	Construction worksites with different hazard types and levels of scene complexity induce different cognitive patterns and cognitive demands and should thus be treated individually.
Liu et al. (2021)	International Journal of Occupational Safety and Ergonomics	30 construction workers	SMI iView X	Visually search hazards in eight given images of site scenes	Fixation time, fixation count, fixation count ratio, fixation time ratio, and mean fixation duration	Paired *t*-test	Semantic cues drive construction workers’ selective attention toward goal-relevant information more effectively, thus improving their hazard recognition performance.
Noghabaei et al. (2021)	Journal of Construction Engineering and Management	30 students	HTC Vive Pro VR headset	Visually search hazards in an immersive virtual construction site	Fixation count, fixation time, mean fixation duration, saccade velocity, and pupil diameter	K-nearest neighbor (k-NN) and support vector machine (SVM)	It is feasible to combine eye-tracking with EEG to detect construction workers’ ability to recognize hazards.
Park et al. (2022)	Safety Science	59 construction workers (supervisors) (53)	Tobii Pro Glasses 2	Visually search hazards in 29 given images of site scenes	Fixation count	Nonparametric *t*-test	Inattentional blindness is responsible for about 50% of the safety hazard perception of construction workers, and safety knowledge nearly does not affect inattentional blindness.

### Device Selection

Various eye trackers have been applied in construction safety studies to measure the visual attention allocation of construction workers. The sampling rate (in Hz), the number of eye images acquired in a second (Leube et al., 2017), is one of the most critical performance parameters of eye trackers (Špakov et al., 2019). A high sampling rate enables the eye trackers to identify and monitor microsaccades, post-saccadic oscillations, and other micro details of eye movement behaviors. However, higher sampling rates often require better eye-tracking sensors and more infrared light sources, increasing experimental cost, and generating more data to be processed (Wierts et al., 2008). Therefore, the researchers should select the sampling rate of the eye trackers according to the specific needs of the study. In eye-tracking studies in construction safety, the sampling rates of selected eye trackers range from 30 to 500 Hz, and most studies selected sampling rates of 50 and 100 Hz. Most of these studies used fixation time and fixation count as the fixation indicators. Eye trackers with a minimum sampling rate of 50 Hz can properly detect the participants’ fixation behavior. Devices used in most eye-tracking studies of construction safety meet the requirements. However, it should also be noted that the low sampling rate tends to create additional variations in the data.

Eye-trackers used in construction safety studies can be categorized into desktop devices and wearable devices. With desktop devices, participants do not need to wear any instrument on the head to capture eye movement data. Participants are asked to sit in front of a monitor and interact with screen-based content. These desktop devices are widely applied in construction safety studies with screen-based stimulus material like images and videos in a laboratory setting (Hasanzadeh et al., 2017a; Jeelani et al., 2017a). These devices facilities to develop AOI compared with wearable devices, which is beneficial to control variables. However, the desktop devices greatly limit the participants’ mobilities, failing to measure construction workers’ eye movements during daily construction activities on an actual construction site (Chong et al., 2021). Wearable devices can address these limitations. Eye-tracking sensors are installed on the glasses and allow participants to walk freely while recording the eye movement data, meeting the requirements of on-site experiments (Cheng et al., 2021; Liao et al., 2021). Some wearable eye trackers can also be integrated with Virtual Reality (VR) headsets and measure eye movement in immersive virtual environments (Kim et al., 2021b; Noghabaei et al., 2021). Because researchers usually cannot expose participants to actual hazards to protect their safety, the ability to integrate with VR significantly extends the application of eye-tracking in construction safety studies (Kim et al., 2021a). However, excessive movements of participants during construction activities tend to cause shifts of glasses and affect the data quality during eye movement data measurement.

### Experiment Task Design

Experiment tasks in eye-tracking studies of construction safety are almost hazard recognition-related ones, in which participants are asked to identify potential hazards from given stimulus material or surrounding environments. Those hazards cover the most common ones at construction sites, such as fall hazards, struck-by hazards, housekeeping hazards, ladder-related hazards, and electrical hazards. Some studies focus on a specific hazard (Li et al., 2019; Kim et al., 2021b), while more studies included multiple hazards (Dzeng et al., 2016; Hasanzadeh et al., 2017b; Han et al., 2020). However, construction workers cannot totally devote themselves to hazard recognition during actual construction activities because their daily work also requires much attention and effort (Chen et al., 2016). To better fit the actual situation in engineering practice, some studies tried to test participants’ hazard recognition performance during simple construction tasks such as walking along a path at a construction site (Hasanzadeh et al., 2018), operating an excavator (Li et al., 2019), and cleaning the road (Kim et al., 2021b). Stimulus materials are the key to experiment design in eye-tracking studies. Various stimulus materials have been adopted in eye-tracking studies of construction safety. Images are the most widely used stimulus materials, which are relatively easy to obtain and carry out experiments under laboratory conditions (Cheng et al., 2021; Liu et al., 2021; Park et al., 2022). However, hazard recognition in images is different from construction workers’ daily work, and therefore hazard recognition performance in images may not be the same as in actual construction sites. In addition, when selecting images, the researchers should pay attention to the irrelevant variables introduced by image factors such as clarity, resolution, and brightness (Han et al., 2020). Some studies directly asked participants to identify potential hazards in actual construction sites (Chong et al., 2021; Liao et al., 2021). This can address the inconsistency between images and actual construction site environments. Selecting construction site environments as stimulus materials enables to test construction workers’ hazard recognition performance during their daily work. However, this will expose construction workers to actual hazards (Hasanzadeh et al., 2018; Jeelani et al., 2018). Experiments at construction sites must ensure the safety of participants. With the development of computer technologies, wearable eye trackers integrated with VR headsets can measure eye movement in immersive virtual environments (Kim et al., 2021b). Therefore, some studies tried conducted experiments in immersive virtual environments, in which participants can realistically and safely perceive and interact with involved objects (Noghabaei et al., 2021). However, the development of realistic VR scenarios, including various hazards, becomes another challenge.

### Area of Interest Determination

The first step in eye movement feature extraction and data analysis is to define the area of interest (AOI). AOIs, defined by the research team, represent items, and locations of interest inside a scene. Many eye-tracking indicators are calculated based on AOIs, and the robustness of AOI-based indicators is vulnerable to the AOI definitions’ accuracy (Bonikowski et al., 2021). In eye-tracking studies of construction safety, AOIs are usually various hazards included in the stimulus materials (Han et al., 2020; Comu et al., 2021). Determination of AOIs usually needs experienced specialists in the subject matter to examine a scene and identify which regions reflect potential hazards to which participants should allocate attention, to maintain situational awareness throughout the scenario (Jeelani et al., 2019; Cheng et al., 2021; Park et al., 2022). It is not difficult to define AOIs when using images as stimulus materials in a controlled laboratory setting since elements in image stimulus materials are static. Nevertheless, AOIs definition will become challenging in an actual construction site or an immersive virtual environment because certain environmental cues such as moving construction machines and workers are dynamic. Even if the stimulus is static, it may be seen differently from different angles by different participants (Cheng et al., 2021). To address these problems, Hasanzadeh et al. (2018) indicated that in eye-tracking studies of construction safety with dynamic stimulus materials such as actual construction sites and immersive virtual environments, AOIs should be defined according to multiple snapshots of participants’ eye movements captured by eye trackers. Selected scenarios should cover all path perspectives seen by most participants and include potential hazards. Most studies just labeled AOIs on the basis of subjective judgements of experts, thus eroding the indicators’ reliability and validity. Data-driven AOI definition methods like fixation clustering help to overcome the lack of subjectivity in conventional AOI pre-definition methods. For example, Xu et al. (2019) developed data-driven AOIs using the output fixation data of Tobii Pro Lab. Specifically speaking, the mean shift approach was applied to cluster all the participants’ fixations for each hazard scenario for each hazard scene. When using this method, the distant threshold in the algorithm should be adjusted to confine the number of clusters. The final number of AOIs within each hazard scenario should be determined based on the principle that the AOIs had clear boundaries and practical meanings. Even so, the data-driven AOIs may be influenced by factors like participants’ cryptic recognition ability and time-varying attention, resulting in the accumulation of potentially unpredictable errors, casting doubt on the blurring of AOI boundaries and the robustness of associated indicators.

### Feature Extraction

Feature extraction is critical to eye-tracking studies of construction safety since appropriate indicators help to decode eye movement information, thus enabling researchers to explore the attention allocation of construction workers during hazard recognition. The eye-tracking indicators in the reviewed studies can be classified into three categories: fixation-derived indicators, saccade-derived indicators, and other indicators. Fixation-derived indicators are the most widely used indicators in eye-tracking studies of construction safety, including fixation time, fixation time ratio, search duration, time to the first fixation, fixation count, fixation count ratio, run count, mean fixation duration, visual attentions index, fixation special density, the accuracy rate of the first fixation, fixation sequence, and intersection coefficient. The major saccade-derived indicator in construction safety research is saccade velocity. Other used eye-tracking indicators include pupil diameter, blink count, and duration. Those major indicators will be discussed as follows.

#### (1) Fixation time and fixation time ratio

Fixation refers to a relatively stationary eye position with a relatively short minimum duration (usually 100–200 ms) (Larsson et al., 2015). Longer fixations indicate a function of participants’ processing objectives. Fixation-derived indicators have been widely used in eye-tracking studies of construction safety to assess the depth of cognitive processing and attention allocation.

Fixation time (FT), also known as dwell time (Hasanzadeh et al., 2018) or fixation duration (Sun et al., 2020), is defined as the total amount of time spent fixating on a particular location, stimulus, or object during a visual search activity (Jeelani et al., 2019). The fixation time for each AOI can thus be calculated as the sum of time of each participant fixating on that AOI, as shown in Equation (1).


(1) $$F\,T_{j}=\sum_{i}\left(E\,\left(f_{\text{ij}}\right)-S\,\left(f_{\text{ij}}\right)\right)$$

in which *FT*_*j*_ is the fixation time for the *j*_th_ AOI, *E*(*f*_ij_) and *S*(*f*_ij_) are the end time and start time for *i*_th_ fixation on the *j*_th_ AOI (Jeelani et al., 2018). The fixation time highlights the speed of information processing and decision-making (Sun et al., 2020). It indicates the concentration level for an AOI and the difficulty degree of tasks (Comu et al., 2021). Therefore, a long fixation time demonstrates that the participant is either attracted to a hazard or confronted by an obstruction in a certain area. This may cause an unclear relationship between participants’ hazard recognition performance and fixation time because researchers cannot ensure whether participants are focusing on a hazard or coming across difficulties during search tasks (Sun and Liao, 2019). For example, Hasanzadeh et al. (2016) reported that decreasing fixation time means higher levels of situation awareness and better hazard recognition performance. However, Jeelani et al. (2019) indicated that participants with better recognition performance spent more time on each hazard. Liao et al. (2019) believed that there is no clear correlation between hazard recognition accuracy and fixation time. Conclusions from these studies are different and even conflicting.

Fixation time ratio (FTR), also referring to dwell percentage, fixation time percentage, and the ratio of on-target, is the percentage of attention allocated to the AOIs rather than to the background (Isaacowitz et al., 2006). It can be calculated by the ratio of the sum of fixation times on AOIs to the total durations of all fixations within the scenario (Area of Glance, AOG). It indicates the amount of visual attention devoted to the hazards relative to the total attention devoted to the scenario in eye-tracking studies of construction safety as shown in Equation (2) (Jeelani et al., 2019).


(2) $$F\,T\,R=\frac{\sum_{i}\left(E\,\left(f_{i}\right)-S\,\left(f_{i}\right)\right)\,i\,n\,A\,O\,I}{\sum_{j}\left(E\,\left(f_{j}\right)-S\,\left(f_{j}\right)\right)\,i\,n\,A\,O\,G}$$

where *E* and *S* represent the end and start time for the fixation on the *i*_th_ AOI or the *j*_th_ AOG. The higher fixation time ratio means the participant allocates more attention to potential hazards instead of other distracting items, which represents better hazard recognition performance (Liao et al., 2019). Hasanzadeh et al. (2017a) proposed different views. They believed construction workers with high hazard recognition performance should allocate their attention throughout the entire scenario to maintain high situational awareness in a dynamic construction workplace. Therefore, they think that a lower fixation time ratio is associated with better hazard recognition performance.

#### (2) Search duration

Search duration is defined as the amount of time a participant spends scanning the workplace to identify potential hazards (Maltz and Shinar, 1999). It represents the attention resource paid by the participants in a scenario (Jeelani et al., 2018). Construction workplaces are usually complicated and involve several hazards. Construction workers are expected to identify all hazards. Still, previous studies have indicated that many workers prematurely end their visual search process after recognizing a few typical hazards, such as trips, falls, and trapped in or between objects, even though some additional hazards may remain unrecognized (Jeelani et al., 2017b; Namian et al., 2018). A longer search duration usually means more effort spent on hazard recognition. Jeelani et al. (2019) reported that construction workers who recognized more hazards spent more time scanning and evaluating the workplace. In other words, a longer search duration usually reflects better hazard recognition performance. Han et al. (2020) used search duration to measure cognitive load for hazard recognition of construction workers. Higher search duration indicates a more complicated scenario for participants, in which they have to spend more attention resources with a higher cognitive load.

#### (3) Time to first fixation

Time to first fixation is another important time-related indicator in eye-tracking studies of construction safety to describe workers’ hazard recognition performance (Sun and Liao, 2019). It refers to the amount of time that passes following the scenario’s first appearance until the participant first fixates on an AOI. It describes how quickly the AOI is fixated. It can be used to reveal which hazards captured workers’ attention more quickly than others (Hasanzadeh et al., 2017b,^2018^). Comu et al. (2021) compared two types of construction safety training methods. In their studies, time to first fixation is applied to assess the adaptation of the participants to the training contents.

#### (4) Fixation count and fixation count ratio

Fixation count is defined as the number of fixations. It is indicative of the importance of the noticeability of areas or objects (Jeelani et al., 2018). A larger fixation count is believed to be associated with superior hazard recognition performance (Hasanzadeh et al., 2017a; Jeelani et al., 2019). More fixation counts also mean participants spend more attention resources during hazard recognition with a higher cognitive load (Han et al., 2020).

Fixation count ratio (FCR) is calculated as the ratio between the number of fixations on a particular object or area and the total number of fixations. In other words, fixation count ratio describes the relative number of fixations within and outside the AOIs as shown in Equation (3) (Jeelani et al., 2019):


(3) $$F\,C\,R=\frac{F\,i\,x\,a\,t\,i\,o\,n\,c\,o\,u\,n\,t\,i\,n\,A\,O\,I}{F\,i\,x\,a\,t\,i\,o\,n\,c\,o\,u\,n\,t\,i\,n\,A\,O\,G}$$

The fixation ratio is found to be predictive of hazard recognition performance (Jeelani et al., 2018). They concluded that construction workers who selectively focus on hazardous areas over other nonhazardous areas demonstrate superior hazard recognition performance. Liu et al. (2021) also indicated that a higher fixation count ratio is usually corresponding to lower distraction and improved search strategy in hazard recognition tasks. However, similar to the fixation time ratio, a high fixation ratio may mean that construction workers have low situation awareness and cannot respond to dynamic changes at construction sites in time (Hasanzadeh et al., 2017a).

#### (5) Run count

Different from fixation count, the run count refers to the number of times a participant’s attention is drawn back to an AOI (Hasanzadeh et al., 2019). Construction workers who have higher run counts return their attention to hazards more frequently (Hasanzadeh et al., 2017b), reflecting better situation awareness and hazard recognition performance (Hasanzadeh et al., 2018). Hasanzadeh et al. (2017a) indicate that this indicator represents the extent to which workers perceived the AOI to be hazardous.

#### (6) Mean fixation duration

Mean fixation duration (MFD), also known as average fixation duration (Liao et al., 2019), refers to the average fixation time of each fixation during a visual search task (Jeelani et al., 2019). It can be calculated by the fixation time divided by the fixation count, as shown in Equation (4) (Jeelani et al., 2018; Noghabaei et al., 2021):


(4) $$M\,F\,D=\frac{F\,i\,x\,a\,t\,i\,o\,n\,t\,i\,m\,e}{F\,i\,x\,a\,t\,i\,o\,n\,c\,o\,u\,n\,t}$$

During a visual search task, a longer mean fixation duration is related to increased levels of attention, visual processing, and cognitive efforts (Jeelani et al., 2019). Jeelani et al. (2018) reported that the longer mean fixation duration represents confusion and difficulty in obtaining information, which could be one of the reasons for low hazard recognition accuracy. However, Jeelani et al. (2019) did not support that the mean fixation duration is predictive of hazard recognition performance of construction workers.

#### (7) Visual attention index

The visual attention index (VAI) quantifies the amount of time spent collecting information from the scene in comparison to the amount of time spent in saccades. It can be calculated by the ratio of fixation time to search duration as shown in Equation (5):


(5) $$V\,A\,I=\frac{F\,i\,x\,a\,t\,i\,o\,n\,t\,i\,m\,e}{S\,e\,a\,r\,c\,h\,d\,u\,r\,a\,t\,i\,o\,n}$$

Because saccades contain little valuable information, a larger visual attention index suggests that the participant spent less time searching and more time processing and comprehending targets (Jeelani et al., 2018).

#### (8) Fixation special density

The construction site usually includes many hazards that are distributed across different locations in the entire workplace. Therefore, the participants are required to scan multiple locations within the workplace to efficiently identify as many hazards as possible. Selective attention to a limited number of locations during hazard recognition may cause poor performance. Fixation special density (FSD) can describe the distribution of attention across the given scenario (Sharafi et al., 2015). Jeelani et al. (2019) proposed a calculation method of fixation special density in eye-tracking studies of construction safety. The work area is divided into subareas using a grid system. The fixation special density can be then calculated by the ratio of the number of cells that received at least one fixation and the total number of cells in the entire work area, as shown in Equation (6):


(6) $$F\,S\,D=\frac{N\,u\,m\,b\,e\,r\,o\,f\,c\,e\,l\,l\,s\,w\,i\,t\,h\,a\,t\,l\,e\,a\,s\,t\,o\,n\,e\,f\,i\,x\,a\,t\,i\,o\,n}{T\,o\,t\,a\,l\,n\,u\,m\,b\,e\,r\,o\,f\,c\,e\,l\,l\,s}$$

#### (9) Accuracy rate of first fixation

The given scenarios of construction sites usually contain both hazards and other background information. Sometimes construction workers cannot pay their attention to hazards firstly. Instead, they are attracted by background information. Accuracy rate of first fixation is defined as the proportion of participants that successfully placed their first fixation in the AOI (i.e., hazards). This indicator describes the distinctness of a hazard or a search target (Han et al., 2020). A larger proportion of participants with their first fixation on the hazard indicates that the hazard can be recognized precisely. Additionally, it indicates that the hazard is more obvious for participants to detect, implying that participants spend fewer attention resources and are under a lower cognitive workload. It is also associated with better hazard recognition performance of construction workers.

#### (10) Fixation sequence

To better describe the visual search pattern and strategy of construction workers, it is necessary to investigate the sequence of where the workers focus on. Fixation sequence is a string that indicates the locations of the fixations generated by a participant in chronological order during the visual search task. Each fixation in the sequence is labeled with the posterior AOI with which it falls. Successful hazard recognition usually follows similar visual search strategies (Xu et al., 2019), and the fixation sequence describes the visual search strategy of construction workers during hazard recognition (Dzeng et al., 2016). There are two forms of fixation sequences to suit different research objectives (West et al., 2006). The extended sequence depicts each fixation point as a letter to construct a string, whereas the collapsed sequence substitutes successive strings made of the same letters with a single letter. For example, the extended sequence “AAABB” can be collapsed to “AB.” The extended sequence shows the full visual search process, while the collapsed sequence emphasizes transitions between AOIs (Liu et al., 2021).

#### (11) Intersection coefficient

Intersection coefficient in the search track is defined as the level of intersection measured by different scan paths crossing each other during the search process (Han et al., 2020). It quantifies the detection complexity in a given scenario. A larger intersection coefficient indicates that the hazards are more complicated or varied. As a result, it becomes more difficult for participants to recognize hazards appropriately. They have to pay more attention resources with a greater cognitive workload.

#### (12) Saccade velocity

Fixations and saccades are two different typical behaviors of eye movements. Saccades, defined as rapid eye movements from one spot to another, vary in duration but typically last for 25–150 ms, depending on the saccade amplitude (Hasanzadeh et al., 2017b). Most of the eye-tracking studies of construction safety remove saccades in eye movement analysis because they think saccades include little valuable cognitive information. However, some studies believe that saccades are necessary for bringing areas outside the focal area into focus or attention, and therefore are critical for visual world navigation. Saccade velocity, usually measured as the average number of pixels traversed by the eyes per unit time during a visual search task, is the most widely used saccade-derived indicator in eye-tracking studies of construction safety (Jeelani et al., 2019). During a visual search activity, a rapid saccade velocity is associated with low levels of arousal and engagement. Additionally, it is related to fatigue and lethargy (Noghabaei et al., 2021).

#### (13) Pupil diameter

Besides fixation-derived indicators and saccade-derived indicators, pupil diameter is another important indicator in eye-tracking studies of construction safety. Changes in pupil diameter indicate emotional arousal and alertness induced by visual detection of sensory stimuli, and pupil diameter rises as a participant processes emotionally engaging stimulus. Kim et al. (2021b) concluded that pupil dilation reflects the increase in cognitive processing information or cognitive load. Liao et al. (2021) also found that hazards can induce pupillary activation of participants. In addition, Li et al. (2019) found that pupil diameter can also be used for assessing mental fatigue. When participants experience mental fatigue, their pupil sizes decrease. They also calculated the percent change in pupil diameter and indicated that participants’ pupil diameter reduction is relatively stable when experiencing mental fatigue.

#### (14) Blink count and duration

Blink count (the number of times eyes blink in the visual search task) and duration (the time of each blink) are also applied in eye-tracking studies of construction safety. Li et al. (2019) found blink behaviors are related to participants’ mental fatigue. Specifically, increased mental fatigue induces an increase in blink count and duration of participants. In other words, blink count and duration may be used to determine whether construction workers are entering the phase of severe mental fatigue.

### Data Analysis Methods

Eye movement data contains rich cognitive information of construction workers, closely related to their safety behaviors. Various data analysis methods have been widely adopted to encode secrets behind eye movement data. Parametric tests such as *t*-test, analysis of variance, are used to compare whether there are significant differences between experimental and control groups (Sun and Liao, 2019). Specifically, using these parametric test methods, researchers examined whether significant differences exist in the eye movement data and hazard recognition performance of construction workers under different conditions, which helps identify the key eye movement indicators or influence factors in construction safety management (Li et al., 2019). For example, Liu et al. (2021) used a *t*-test to assess the differences in hazard recognition performance between the construction workers with and without semantic cues. Comu et al. (2021) used analysis of variance to investigate the interaction between construction worker group type and safety training methods. However, these parametric tests usually have strict testing criteria for the sample data, such as normal distribution, a random sample from a population, and quality of variances. The experimental eye movement data are usually small samples and cannot satisfy these criteria (Anderson, 2001). Non-parametric tests can deal with data that do not meet the assumption of normal distribution (Park et al., 2022). For example, Kim et al. (2021b) used the Mann-Whitney *U* test to assess the significant differences in pupil diameter changes between two kinds of behavioral responses. Furthermore, the random permutation test, using the actual data instead of ranking it in other non-parametric tests, is considered an alternative technique to address these problems (Hasanzadeh et al., 2018). Its basic idea is to construct a reference distribution by recalculating data statistics through resampling (Berger, 2000). In other words, the permutation tests calculate the probability of reaching a value equal to or greater than the observed value of a test statistic after randomly shuffling data several times (Ernst, 2004). This method can offer higher power despite the non- and mixed-normality of the distributions across groups and the limited sample size (Hasanzadeh et al., 2017b). Additionally, it aids in getting more robust results when outliers and missing data are present. Thus, the permutation test technique has been adopted in many eye-tracking studies of construction safety (Hasanzadeh et al., 2019; Liao et al., 2021). Analysis of variance does not consider the relationships between variables, either (Hasanzadeh et al., 2017a). Discriminant analysis can address this limitation since the analysis is carried out on the basis of the variable interactions. The derived function can also be used to predict the hazard recognition performance of construction workers based on eye movement data (Sun and Liao, 2019). Correlation analysis is another widely used multivariate statistical analysis method in data analysis in eye-tracking studies of construction safety. Correlation analysis is often used to investigate correlations between variables, such as correlations between eye-tracking indicators and hazard recognition performance (Li et al., 2019), neural activity (Liao et al., 2021), and worker personalities (Hasanzadeh et al., 2019).

Investigating the relationship between eye-tracking indicators and hazard recognition performance of construction workers is a typical pattern recognition task, aiming at mining the rich cognitive information contained in eye movement data. The hazard recognition performance prediction based on eye movement data can be regarded as supervised learning tasks, in which various machine learning models can be applied to develop accurate classifiers. Regression analysis is the simplest and most widely used machine learning model in eye-tracking studies of construction safety to predict hazard recognition performance of construction workers based on eye movement (Jeelani et al., 2017a; Jeelani et al., 2019; Liao et al., 2019). In addition, eye movement data can also be used to develop various other machine learning-based classifiers. For example, Noghabaei et al. (2021) combined EEG data and eye movement data and developed two different machine learning classifiers, the *k*-nearest neighbor model and support vector machine model, to predict the hazard recognition performance of construction workers. They indicate that the support vector machine model can reach an accuracy of around 93% and has better performance. Kim et al. (2021b) developed a support vector machine model for inattentiveness to struck-by hazards of construction workers based on ECG and eye-tracking indicators.

In addition to traditional statistical methods and machine learning models, it can be seen that qualitative comparative analysis (QCA) is also used in eye-tracking studies of construction safety. This method combines the advantages of qualitative and quantitative research methods to allow systematic comparison of a limited number of samples (Chong et al., 2021), which is highly applicable in revealing the influence of complex relationships among multiple antecedents on the results. This method can identify the common visual patterns of successful hazard recognition (Cheng et al., 2021).

### Main Findings

Eye-tracking techniques provide a powerful tool to explore visual attention allocation of construction workers during hazard recognition (Jeelani et al., 2017a). Previous studies proved that construction workers’ hazard recognition performance is closely related to both workplace conditions (Liao et al., 2019) and construction workers’ personal attributes. As for workplace conditions, brightness, distinctiveness, and tidiness of construction sites all affect the hazard recognition performance of workers (Han et al., 2020). More distinct hazards and a tidy site reduce construction workers’ cognitive loads and improve their performance in hazard recognition, while site brightness has positive and negative effects. Construction workers’ personal attributes, such as work experience (Dzeng et al., 2016; Comu et al., 2021), personalities (Hasanzadeh et al., 2019), fatigue level (Li et al., 2019), safety knowledge (Park et al., 2022), etc., also significant affect their hazard recognition performance. In addition, it is found that successful hazard recognitions have similar visual search patterns (Xu et al., 2019). For example, electricity-related hazards should be recognized based on object identification, while struck-by hazards should be identified based on the objects and their pivot points or probable movement trajectories (Chong et al., 2021). Navigated visual pattern help to improve hazards recognition effectiveness (Liu et al., 2021). Main findings from previous studies provide valuable references for construction safety training and on-site management.

## Discussion

### Implications

The construction sites are hazardous and dynamic, and construction workers are exposed to various potential hazards during their daily work. Safety management has become the most critical concern in the construction industry. In the realm of construction safety research, visual attention is important since hazards recognition is a complicated and multidimensional cognitive process that requires proper attention allocation of construction workers. Eye-tracking has been flourishing in recent studies of construction safety because it serves as the most direct measure of visual attention of construction workers. In addition, eye-tracking is a high temporal-resolution technique, which is suitable for capturing the fast-changing cognitive information of construction workers in a dynamic workplace.

When designing an eye-tracking experiment, the researchers should recruit participants with enough size and representativeness. These participants are usually asked to identify potential hazards from given screen-based stimulus materials, construction sites, or even immersive virtual environments. The device selection should consider the stimulus materials used. Desktop eye trackers are suitable for screen-based materials such as videos and images, while wearable eye trackers are suitable for actual construction sites. Some eye trackers can be integrated with VR headsets. AOI determination is important for eye-tracking data analysis. AOIs are usually determined by the judgment of experts or data-driven algorithms. Fixation-derived indicators, saccade-derived indicators, and some other indicators are usually used in eye-tracking studies of construction safety. Traditional statistics models, machine learning models, and QCA have good performance in eye movement data analysis in eye-tracking studies of constriction safety.

Eye-tracking techniques provide a powerful tool for investigating construction workers’ attention allocation, laying the foundation for incorporating neuroscience into construction safety management. Firstly, eye-tracking studies can identify better visual patterns during hazard recognition, which helps construction workers to improve their visual search strategies and hazard recognition performance. It can also provide objective feedback on construction workers’ performance and training effects during construction safety training. This information aids in developing more effective and intelligent safety training systems. In addition, previous studies identified key influence factors in construction workers’ hazard recognition performance. They indicated that construction workers’ hazard recognition performance is closely related to their own characteristics, environmental conditions, and visual search strategies, which provides construction managers valuable references for enhancing safety management.

### Limitations and Recommendations

Because the application of eye-tracking techniques in construction safety is still in the initial stage, there are some limitations in existing studies, including imperfect experiment design, unavailability to authoritative open-access datasets, unclear relationship between visual attention and brain activities, imperfect data analysis models, and unavailability to research at the group level. Recommendations for future research are also proposed as follows.

Firstly, the experiment design still has many shortcomings. For example, most studies selected students as participants instead of construction workers but they cannot validate whether the drawn results and conclusions also suit construction workers. In addition, most of these studies failed to assess construction workers’ hazard recognition performance during their daily work. The authors need to further improve their experiment design to make it reveals the real construction activities as much as possible. For example, researchers should try to recruit construction workers as participants and not just students.

Secondly, an authoritative open-access eye-tracking dataset of construction workers is not available. Although there have many studies measuring and recording the eye movement of construction workers during hazard recognition, the researchers all did not publish and share their data. The unavailability of authoritative open-access datasets makes it difficult to objectively compare the accuracy and efficiency of various data analysis models because their experimental conditions and datasets are not the same. This hinders the further improvement and development of related algorithms. There are many internet platforms for researchers to share their datasets, and many academic journals can also publish papers along with the accompanying datasets. In fact, we can find many famous open-access eye-tracking datasets in other research fields. Therefore, the researchers are suggested to share parts of their data in future research to develop an authoritative open-access eye-tracking dataset of construction workers, which facilitate researcher to further improve data analysis tools.

Thirdly, the relationship between visual attention and brain activities of construction workers during hazard recognition is unclear. Although there have been some studies integrating eye-tracking techniques with other techniques that capture brain activities during the recognition process, such as EEG and fNIRS, these studies failed to explain the relationship between visual attention and brain activities. In fact, the visual attention of construction workers is affected and even controlled by their brain activities. The researchers are suggested to clarify the relationship between visual attention and brain activities, which allows the mechanism of individual attention allocation to be better understood.

Fourthly, the data analysis tools are either not perfect. Although machine learning models are good at processing abundant data but not yet fully applied in eye-tracking studies of construction safety. Traditional machine learning models are not capable of learning on their own because their performance is largely dependent on hand-designed features. Thus, deep learning models that can learn features and perform classification have emerged. Deep neural networks may obtain excellent results on many tasks such as classification, regression, and generation by using manually derived features as model inputs or simply using raw data as inputs. Nevertheless, deep learning models have not been applied in eye-tracking studies of construction safety. Future studies can consider using mainstream deep learning models to improve the model performance in eye-tracking studies of construction safety (Klaib et al., 2021). In addition, data analysis in existing eye-tracking studies of construction safety is conducted offline. In other words, the researchers firstly record the eye movement data and then download it for further analysis. Existing data analysis tool may not be capable to handle the large amount of real-time eye movement data. Therefore, it is of great significance to develop a model that can monitor attention allocation of construction workers real-time in future research, which can identify unsafe individuals and behaviors, thus issuing an early warning in time.

Lastly, the existing studies failed to investigate construction workers’ behaviors at the group level. It is noted that all exiting eye-tracking studies of construction safety investigated construction workers’ behaviors at the individual level. However, construction activities are usually organized by groups. Factors at the group level such as safety climate and relationships among group members also influence their behaviors. Researchers are recommended to further investigate the key factors in construction safety at the group level with eye-tracking techniques in the future.

## Conclusion

The eye-tracking technique serves as a promising tool for enhancing construction safety management from the perspective of neuroscience. This manuscript systematically reviewed eye-tracking studies of construction safety, including studies that validated the potential of eye-tracking techniques as a measure of attention allocation of construction workers during hazard recognition. It summarized the major issues in eye-tracking studies of construction safety, including participant selection, device selection, task design, feature extraction, and data analysis. Researchers, particularly those who are new to the discipline, may utilize it as a systematic guide to learn how to conduct eye-tracking research of construction safety. Findings summarized in this review provide construction managers valuable references for enhancing safety management. The present review highlighted the limitations in previous studies, including imperfect experiment design, unavailability to authoritative open-access datasets, unclear relationship between visual attention and brain activities, imperfect data analysis models, and unavailability to research at the group level. Therefore, future studies are needed to expand further the current knowledge based on the relationship between visual attention and hazard recognition, allowing practitioners to improve construction worker performance while keeping on-site hazards under control.

## Author Contributions

BC: conceptualization, methodology, data analysis, writing−original draft, and funding acquisition. XL: conceptualization, data analysis, writing−review and editing, funding acquisition, and supervision. XM: methodology, data analysis, and writing−original draft. HC: data analysis, writing−original draft, and funding acquisition. JH: conceptualization, methodology, writing−review and editing, funding acquisition, and supervision. All authors contributed to this article significantly during its preparation.

## Conflict of Interest

The authors declare that the research was conducted in the absence of any commercial or financial relationships that could be construed as a potential conflict of interest.

## Publisher’s Note

All claims expressed in this article are solely those of the authors and do not necessarily represent those of their affiliated organizations, or those of the publisher, the editors and the reviewers. Any product that may be evaluated in this article, or claim that may be made by its manufacturer, is not guaranteed or endorsed by the publisher.
